# Impact of contrast agents on organ dosimetry in paediatric diagnostic fluoroscopy: the upper gastrointestinal series

**DOI:** 10.1088/1361-6560/ae36e3

**Published:** 2026-01-28

**Authors:** Wyatt W Smither, David Borrego, Kimberly Applegate, Wesley E Bolch, Emily L Marshall

**Affiliations:** 1 J. Crayton Pruitt Family Department of Biomedical Engineering, University of Florida, Gainesville, FL 32611, United States of America; 2 Center for Science and Technology, US Environmental Protection Agency, Washington, DC 20460, United States of America; 3 Department of Radiology, University of Kentucky, Lexington, KY 40536, United States of America; 4 Department of Radiology, University of Florida, Gainesville, FL 32611, United States of America

**Keywords:** paediatric fluoroscopy, organ dosimetry, ICRP phantoms, dose coefficients, upper gastrointestinal examination

## Abstract

*Objective.* Barium and iodinated contrast media are ubiquitous with upper gastrointestinal (UGI) series examinations performed on paediatric patients. The present study quantifies the impact of contrast media on organ absorbed and detriment-weighted doses for UGI examinations. *Approach.* A paediatric radiologist and a medical physicist created reference imaging fields for four complete UGI series examinations encompassing the newborn and 1-year-old female for both normal and abnormal disease states. Monte Carlo radiation transport simulations were performed for these four cases, with and without contrast media, using the international commission on radiological protection’s voxel-based reference computational phantoms. *Main results.* Estimates of detriment-weighted dose and absorbed doses to the colon, heart wall, kidneys, lungs, small intestine wall, spleen, stomach wall, thymus, thyroid, and remainder tissues are reported. For fields with contrast media the organ absorbed doses and detriment-weighted dose decreased by up to 50% and 26%, respectively, with the dose for the complete examination, i.e. not per field, decreasing by up to 26% for organs impacted by the presence of contrast media. *Significance.* Overall, relative doses were shown to decrease for simulations that included contrast media due to selective absorption of the x-ray beam by the contrast media. This study, however, did not investigate the effects of the automatic brightness control which could result in organ absorbed doses increasing due to compensation by the fluoroscopy machine when contrast media is present in the field.

## 1. Introduction

The international commission on radiological protection (ICRP) established Task Group 113 under the guidance of ICRP Committee 2 (Dosimetry) and Committee 3 (Medicine) to provide reference organ and effective dose coefficients (DCs) for common diagnostic imaging examinations in radiography, fluoroscopy, and computed tomography. These DCs are assembled following detailed Monte Carlo radiation transport simulations using the ICRP voxel-based reference computational phantoms (VRCPs). To this end, this subgroup is tasked with defining reference imaging examinations and computing organ absorbed and detriment-weighted DCs for common paediatric diagnostic fluoroscopy examinations for both normal and abnormal clinical outcomes. The detriment-weighted dose is defined as the individual, sex-specific contribution to effective dose as defined in ICRP Publication 103 (ICRP 2007). Use of the detriment-weighted dose instead of the effective dose in the current work reflects the clear differences seen in fluoroscopic examinations on patients of different sex. Paediatric diagnostic fluoroscopy procedures remain relatively common with the upper gastrointestinal (UGI) series being the most commonly performed procedure (Dorfman *et al* 2011, NRCP 2019). Infants and children under the age of 2 years are more likely to undergo a UGI series examination compared to older children as conditions warranting the examination are often congenital. While the use of upper endoscopy has increased over the past two decades, the procedure typically requires sedation and may be less accessible to regions of low- or middle-income patient populations. The UGI series examination is relatively low risk, but the examination involves exposing paediatric patients to ionizing radiation which must be communicated to the referring clinicians and to the patient’s family. It is important to justify these examinations and to have appropriately trained staff to perform radiology examinations that are optimized.

Our earlier work (Smither *et al* 2024) demonstrated that derived DCs may vary by as much as 22% when iodinated contrast media is explicitly modeled in radiation transport simulations that recreate clinical voiding cystourethrogram examinations. The present work calculates organ absorbed and detriment-weighted doses and total doses for paediatric UGI series examinations to quantify the dosimetric impact of both barium and iodinated contrast agent inclusion in such examinations. To accomplish this aim, radiation transport simulations, using Monte Carlo techniques, modeled the UGI series examination with and without administered contrast agents for normal and abnormal diagnoses on the ICRP female newborn and 1 year-old VRCPs.

## 2. Methods

### 2.1. Creation of the UGI outlines

**Figure 1.**
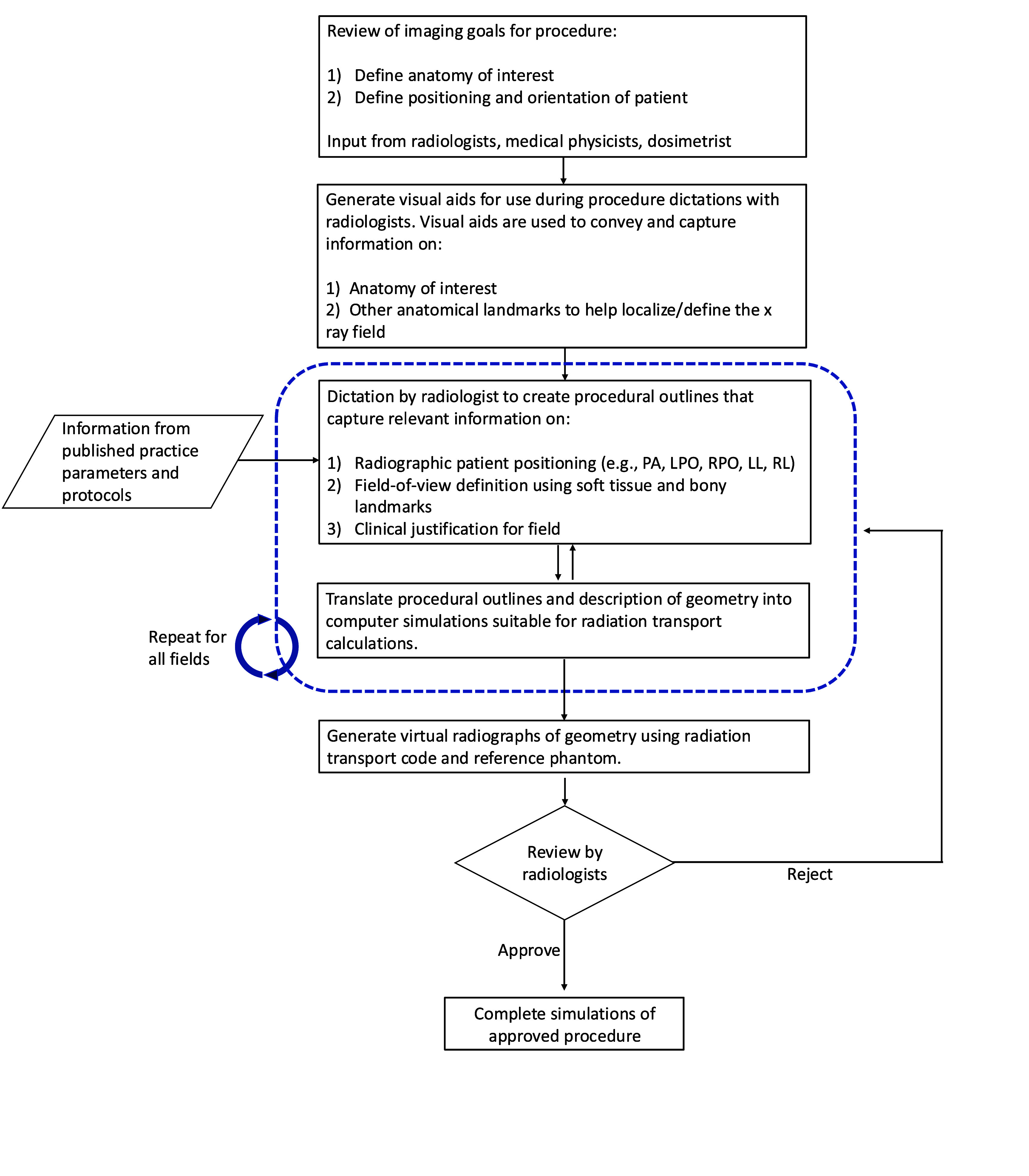
Workflow of procedure outline development for the UGI series examinations. This figure is slightly adapted from Smither *et al* (2024). © 2024 Institute of Physics and Engineering in Medicine. All rights reserved.

The ICRP female newborn and 1 year-old VRCPs were used to create reference imaging fields for outpatient and inpatient care scenarios; the latter encompassing an abnormal clinical diagnosis. Reference imaging fields were defined according to the American College of Radiology practice parameters (ACR 2025), Image Gently^5^5www.imagegently.org/Procedures/fluoroscopy. principles on the use of fluoroscopy, and with the help of a paediatric radiologist and medical physicist with a combined 40 years of clinical experience. For normal clinical diagnosis, the reference imaging fields were defined to use conventional barium contrast media whereas for emergent cases, typical of inpatient care, reference imaging fields model iodinated contrast media administered via a nasogastric or orogastric tube. In total, nine imaging fields for the normal UGI series scenario and seven for the abnormal UGI series scenario were created. Imaging procedure outlines for the UGI series were created using a standardized workflow shown in figure 1 under the assumption that a general adult radiologist would be performing the procedure, which resulted in demarcated field margins that are less tightly collimated when compared to how a highly specialized paediatric radiologist may perform the examination.

**Figure 2.**
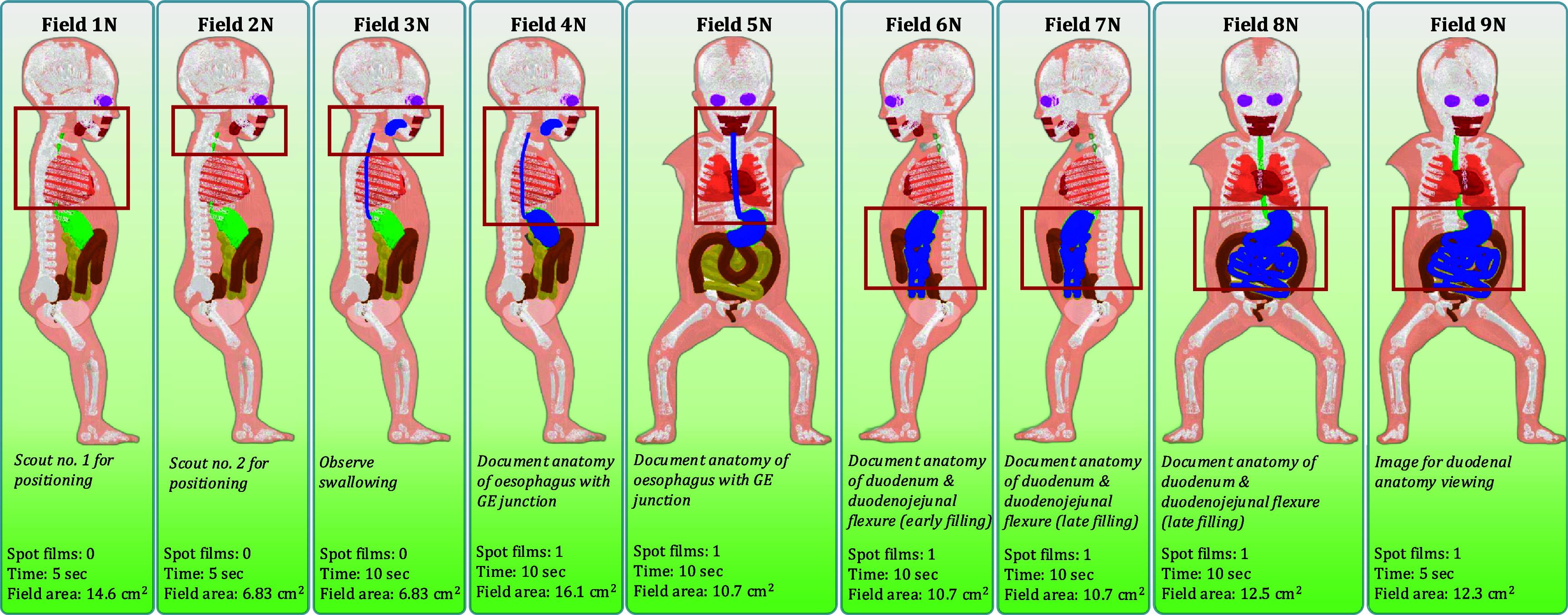
Examination outline with demarcated fields for a newborn female undergoing a UGI series resulting in a normal diagnosis. Field area calculated at 20 cm from the x-ray source.

**Figure 3.**
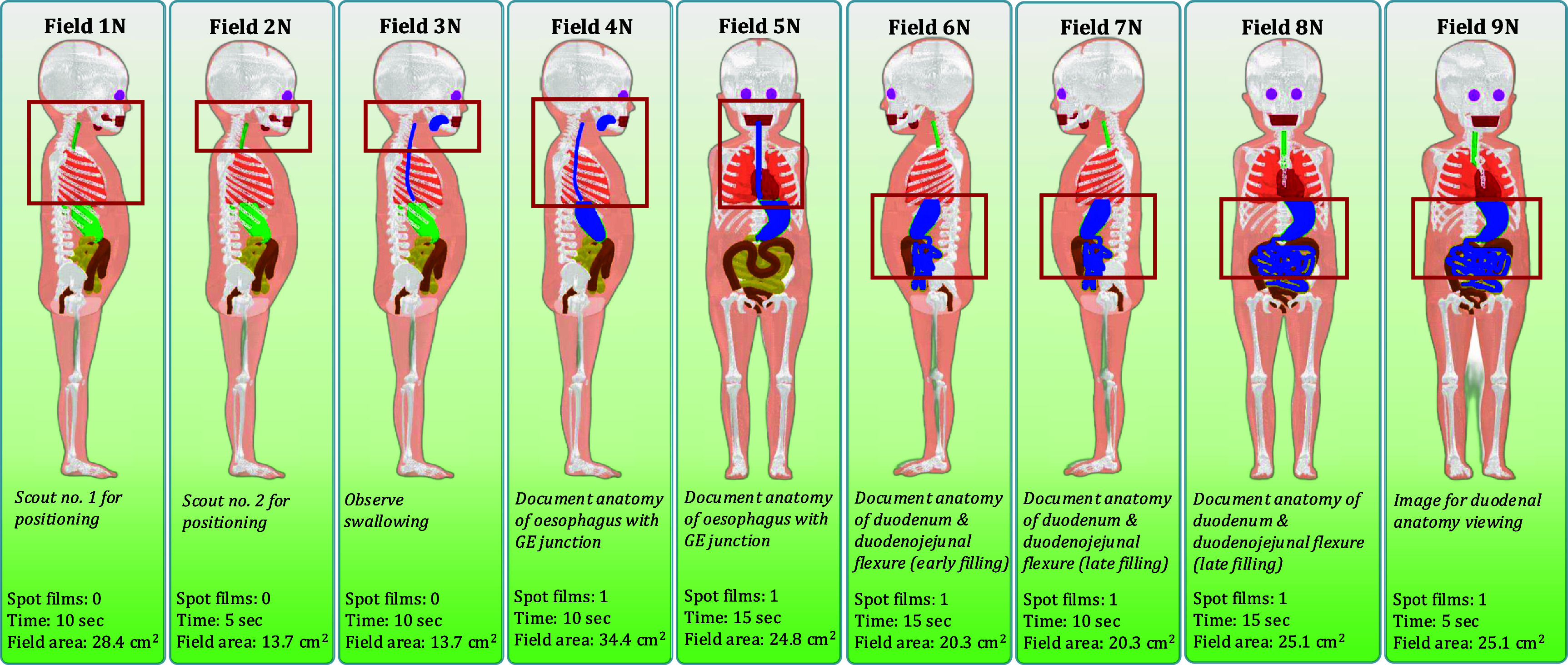
Examination outline with demarcated fields for a 1 year-old female undergoing a UGI series resulting in a normal diagnosis. Field area calculated at 20 cm from the x-ray source.

For the UGI series examination resulting in a normal diagnosis, fields of view and descriptions of anatomical investigation are the same for the newborn and 1 year-old female. The required fluoroscopy time is 75 s and 95 s for the newborn and 1 year-old females, respectively. The first field in this series, Field 1 N (No. 1—Normal diagnosis), is an initial broad scout for review of anatomy and positioning over the oropharynx and chest with the patient in the left lateral position. Field 2 N is a second scout with a tighter x-ray beam collimation focusing the imaging field on the pharynx and oesophagus. Field 3 N is identical to Field 2 N apart from barium contrast media being included in the mouth to initiate the observation of swallowing. Field 4 N expands the field of view to document the gastroesophageal junction. Field 5 N positions the patient in the posterior-anterior position to document the gastroesophageal junction. Field 6 N has the patient in a right lateral position to document duodenum and duodenojejunal junction anatomy during early filling. Field 7 N has the patient in the same configuration as Field 6 N but documents anatomy during late duodenal filling. Field 8 N has the patient in a posterior-anterior position to document late emptying of stomach and to observe the duodenojejunal junction. Field 9 N has the patient in a shallow (15°) left posterior oblique position to view the duodenum and proximal small intestine. Radiographic spot films are taken with the same field margins as the fluoroscopy component during Fields 4 N, 5 N, 6 N, 7 N, 8 N, and 9 N. Resulting field margins for these normal UGI series examinations are shown in figures 2 and 3.

**Figure 4.**
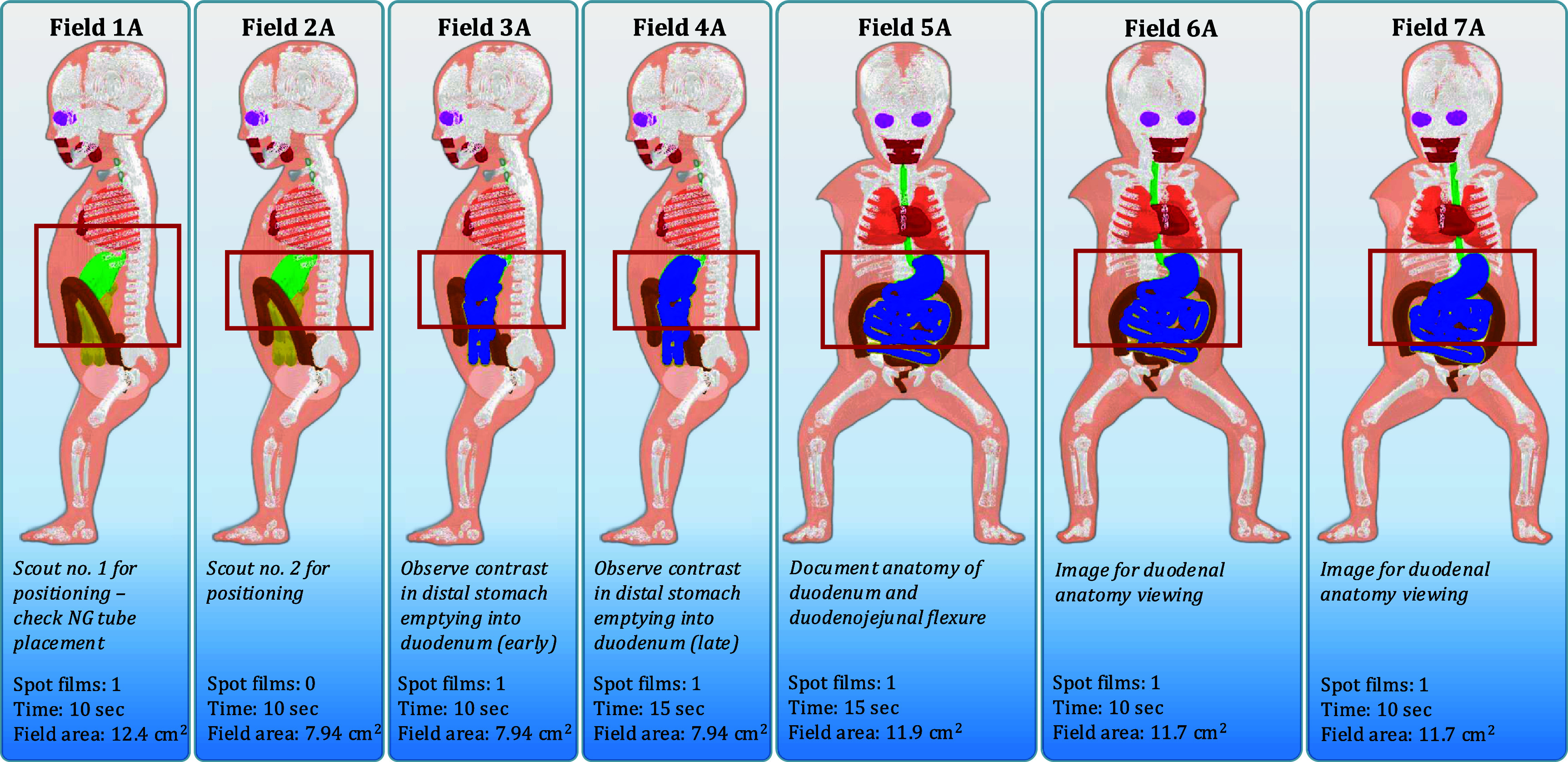
Examination outline with demarcated fields for a newborn female undergoing a UGI series resulting in an abnormal diagnosis. Field area calculated at 20 cm from the x-ray source.

**Figure 5.**
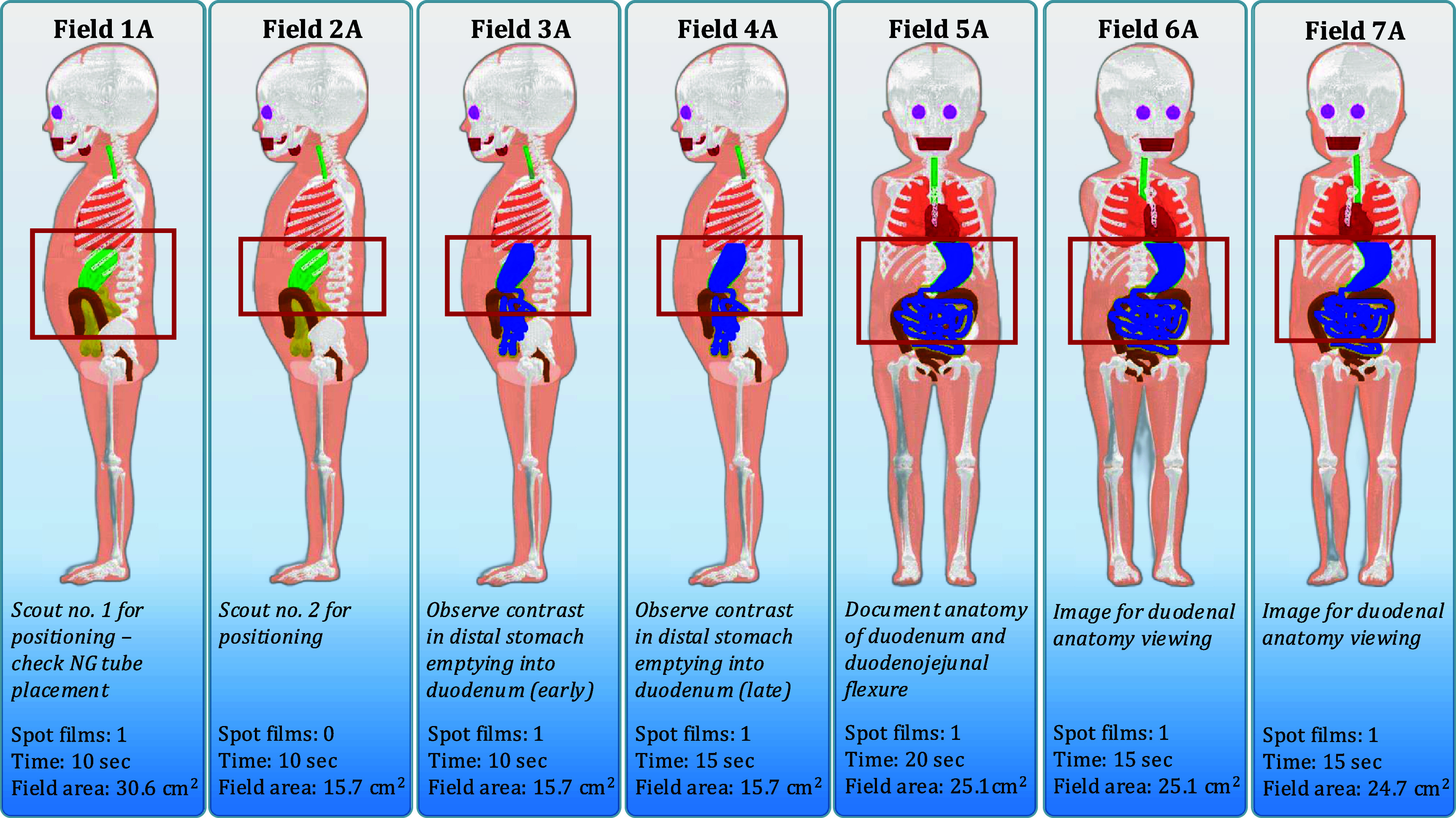
Examination outline with demarcated fields for a 1 year-old female undergoing a UGI series resulting in an abnormal diagnosis. Field area calculated at 20 cm from the x-ray source.

For the abnormal or emergent situation, the UGI series examination is performed using a nasogastric or orogastric tube to ensure the iodinated contrast media is injected directly into the stomach. The required fluoroscopy time is 80 s and 95 s for the newborn and 1 year-old females, respectively. The abnormal scenario is the most important clinical diagnosis for the radiologist to make in these two age groups: either malrotation with volvulus or Ladd’s bands for both age groups. Field 1 A (No. 1—Abnormal diagnosis) is to check tube position and for bowel distention with a large field of view to include the patient’s chest and abdomen. Field 2 A is the same position as Field 1 A with a tighter x-ray beam collimation at the level of the stomach and upper abdomen. Field 3 A has iodinated contrast media in the stomach to investigate early emptying into the duodenum with the patient in a right lateral position. Field 4 A is the same position as Field 3 A to view late stomach emptying. Field 5 A has the patient in a posterior-anterior position to document the duodenal junction as well as showing the upper abdomen. Field 6 A has the patient in a shallow (15°) left posterior oblique position for visualization of the duodenum. Field 7 A has the patient in a shallow (15°) right posterior oblique position for visualization of the duodenum. Radiographic spot films are taken with the same field margins as the fluoroscopy component during Fields 1 A, 3 A, 4 A, 5 A, 6 A, and 7 A. Resulting field margins for these abnormal UGI series examinations are shown in figures 4 and 5.

### 2.2. ICRP reference paediatric computational phantoms

The ICRP has developed a series of ten paediatric reference computational phantoms for male and female newborn, 1 year-old, 5 year-old, 10 year-old, and 15 year-old for the purposes of estimating organ absorbed and detriment-weighted doses from exposures to radiation (ICRP 2020). In this study, the ICRP newborn and 1 year-old female paediatric voxel reference computational phantoms were used with their arms removed to be representative of the clinical practice of having the patient’s arms rotated outside of the x-ray beam to avoid having them appear in the x-ray image. These phantoms are versatile in that they allow free modification of material composition, such as adding contrast media, and modification of anatomy. Reference morphometry values for the virtual patient models are provided in table 1.

**Table 1.** Reference height, mass, and surface area for the ICRP female newborn and 1 year-old voxel computational phantom.

Phantom	Height (cm)	Mass (kg)	Surface area (m^2^)
Newborn female	51	3.5	0.24
1 year-old female	76	10	0.48

### 2.3. x-ray spectrum selection

At UF Health in Gainesville, Florida, common x-ray technique factors for UGI procedures performed on newborns and 1 year-olds were found to have peak tube potentials of 65 kVp with a half-value-layer (HVL) of 4.41 mm Al and 85 kVp with a HVL of 3.22 mm Al for the fluoroscopy and radiographic spot film components, respectively. Six unique x-ray spectra were generated using the SPEKTR 3.0 toolkit (Punnoose *et al* 2016). SPEKTR 3.0 is a physics-validated MATLAB^6^6www.mathworks.com/products/matlab.html. toolkit library that iteratively computes x-ray spectra for peak tube potentials between 20 and 150 kVp in 1 kV bins based on the tungsten anode spectral model using an interpolating cubic splines algorithm (Hernandez and Boone 2014). Details on x-ray spectra peak tube potential, HVL, and mean energy are given in table 2. Organ absorbed and detriment-weighted DCs for the six generated spectra were linear (HVL) and log-linear (kVp) interpolated to determine DCs for the fluoroscopy and spot film x-ray technique factors used in routine clinical practice at UF Health.

**Table 2.** Characteristics of the x-ray spectra used in this study.

Peak tube potential (kVp)	HVL (mm Al)	Average energy (keV)
60	2.5000	38
60	5.7448	47
80	2.9000	44
80	7.7020	57
110	3.9000	52
110	9.9905	69

### 2.4. Contrast material

Contrast media for UGI series examinations were modeled after Bracco Diagnostics’ Liquid E-Z-PAQUE® oral suspension (barium sulfate 60% w/v)^7^7www.bracco.com/us-en-2020-12-03-spc-liquid-e-z-paque. for the normal UGI series and GE Healthcare’s OMNIPAQUE™ (iohexol) Injection (140 mg iodine ml^−1^)^8^8www.gehealthcare.com/-/jssmedia/8a5df56afa4f4ea78352d110b227e956.pdf. for the abnormal UGI series. Chemical formulae, molecular weights, and fractional mass of constituent parts for these contrast media are provided in tables 3 and 4.

**Table 3.** Liquid E-Z-PAQUE® oral suspension (barium sulfate 60% w/v) contrast agent details.

Compound name	Molecular formula	Molecular weight (g mol^−1^)	Fractional mass of solution
Barium sulfate	BaSO_4_	233.3880	0.4098
Water	H_2_O	18.0146	0.5902

**Table 4.** OMNIPAQUE™ 140 mg iodine ml^−1^ (302 mg of Iohexol ml^−1^) contrast agent details. Edetate calcium disodium and tromethamine are inactive ingredients used for stabilizing the OMNIPAQUE™ solution.

Compound name	Molecular formula	Molecular weight (g mol^−1^)	Fractional mass of solution
Iohexol	C_19_H_26_I_3_N_3_O_9_	821.1238	0.2595
Edetate calcium disodium	C_10_H_12_CaN_2_Na_2_O_8_	374.2676	0.0001
Tromethamine	C_4_H_11_NO_3_	121.1338	0.0010
Water	H_2_O	18.0146	0.7394

During a clinical UGI series examination, changes in the patient’s mouth, oesophagus, stomach, and small intestine volumes would be seen throughout the procedure as contrast media moves through a patient’s GI system. The ICRP paediatric VRCPs have organs that are rigid structures that cannot change volume throughout the examination like a patient’s normal, motile GI system. In addition, there are no unique anatomical identifiers for the mouth, hypopharynx, oesophageal lumen, or the duodenum separate from the entire small intestine. To overcome this limitation and still estimate the impact of a contrast-filled upper GI system, contrast media had to be placed strategically. As there is no oral cavity to represent the mouth, contrast volume is mixed with the tongue muscle in the form of tongue oral mucosa and inner tongue organ regions (i.e. when referring to both, reference tongue material). Additionally, there is no oesophageal lumen present within the ICRP paediatric VRCPs due to limitations in the phantom voxel resolution and therefore contrast material had to be mixed with the entire volume of the oesophagus, which was originally intended to be representative of the oesophageal wall. Contrast was also mixed with stomach and small intestine contents in various proportions to approximate contrast movement from the stomach to the small intestine. The mixture of contrast material and organ tissue is achieved by directly modifying the region’s material composition to reflect an appropriate combination of the original organ material and the contrast media. Distributions and proportions by volume of organ contents and contrast media for a normal UGI series examination for both newborn and 1 year-old female phantoms are shown in table 5. Distributions and proportions by volume for organ contents and contrast media for an abnormal UGI examination for both newborn and 1 year-old female phantoms are shown in table 6. Elemental compositions for organ regions containing contrast media for each field are found in annex A.

**Table 5.** Distribution of contrast material per organ within the newborn and 1 year-old ICRP VRCPs undergoing a UGI procedure resulting in a normal diagnosis. ‘30% E-Z-PAQUE/70% mouth’ refers to 70% by volume being reference tongue material composition and 30% by volume being Bracco diagnostics’ liquid E-Z-PAQUE® oral suspension (see table 3). The same method is applied to the oesophagus (whole organ), stomach contents, and small intestine content materials.

Field	Mouth	Oesophagus	Stomach	Small intestine
1	—	—	—	—
2	—	—	—	—
3	30% E-Z-PAQUE®/70% Mouth	100% E-Z-PAQUE®	—	—
4	10% E-Z-PAQUE®/90% Mouth	100% E-Z-PAQUE®	10% E-Z-PAQUE®/90% Contents	—
5	—	100% E-Z-PAQUE®	40% E-Z-PAQUE®/60% Contents	—
6	—	—	100% E-Z-PAQUE®	10% E-Z-PAQUE®/90% Contents
7	—	—	90% E-Z-PAQUE®/10% Contents	20% E-Z-PAQUE®/80% Contents
8	—	—	90% E-Z-PAQUE®/10% Contents	20% E-Z-PAQUE®/80% Contents
9	—	—	90% E-Z-PAQUE®/10% Contents	20% E-Z-PAQUE®/80% Contents

**Table 6.** Distribution of contrast material per organ within the newborn and 1 year-old ICRP VRCPs undergoing a UGI procedure resulting in an abnormal diagnosis. ‘80% OMNIPAQUE™/20% Contents’ refers to 20% by volume being the reference stomach contents material composition and 80% by volume being GE healthcare’s OMNIPAQUE™ injection (see table 4). The same method is applied to the small intestine content material.

Field	Stomach	Small intestine
1	—	—
2	—	—
3	100% OMNIPAQUE™	10% OMNIPAQUE™/90% Contents
4	80% OMNIPAQUE™/20% Contents	30% OMNIPAQUE™/70% Contents
5	80% OMNIPAQUE™/20% Contents	30% OMNIPAQUE™/70% Contents
6	80% OMNIPAQUE™/20% Contents	30% OMNIPAQUE™/70% Contents
7	80% OMNIPAQUE™/20% Contents	30% OMNIPAQUE™/70% Contents

### 2.5. Monte Carlo radiation transport simulations

In this study, the particle and heavy ion transport code system (PHITS) version 3.35 (Sato *et al* 2024), a benchmarked (Iwamoto *et al* 2022) general-purpose Monte Carlo particle transport simulation code, was used with the EGS5 cross section library for photon and electron transport (Hirayama *et al* 2005). The x-ray source was defined as a cylindrical source with zero height and radius to be indicative of an anisotropic point source. Source solid angle was defined depending on the field size calculated by an in-house MATLAB script to create a conical ray of x-ray source photons. Physical lead collimators with zero particle importance were placed 10 cm away from the source with an appropriately sized rectangular air window that shaped the conical beam to a rectangular diverging field. Secondary particle tracking was turned on. Photons and electrons were tracked until they fell below 2 and 20 keV, respectively, after which their remaining energy was deposited locally. Absorbed doses were calculated in units of Gy/source using the T-Deposit tally in PHITS for the following organ sets in the newborn and 1 year-old female VRCPs: colon, heart wall, kidneys, lungs, small intestine wall, spleen, stomach wall, thymus, thyroid, remainder tissues defined by ICRP Publication 103 (ICRP 2007), and the detriment-weighted dose. A second simulation was run with the same x-ray beam geometry without a computational phantom present and with secondary particle tracking turned off; kerma was tallied in an air volume and field area was calculated at 20 cm from the source for the creation of DCs by taking the ratio between organ absorbed or detriment-weighted dose and kerma-area product. The radiation transport simulations were run on the University of Florida’s supercomputer, HiPerGator, which has 19 200 AMD EPYC 9655P Turin 2.5 GHz (4.5 GHz Boost) cores. PHITS simulations were run with 5000 000 histories in 20 batches with no variance reduction, tally filtering, or denoising techniques employed. The simulation terminated when either 100 million total histories or a wall time of 24 h was reached. Updated fluence-to-dose response functions (DRFs) for red (active) bone marrow and bone endosteum were employed within the PHITS simulation where the DRF as a function of traversing particle energy was log–log interpolated and applied to report DCs for red bone marrow and the bone endosteum (ICRP n.d.).

In order to quantify the effect of including contrast media on organ absorbed and detriment-weighted doses in these Monte Carlo radiation transport simulations, two sets of simulations were run for each unique phantom and disease state combination: one complete series with contrast included in the appropriate organs and time points and one complete series without contrast in any organ. Ratios between the two were computed and reported with changes being discussed in reference to the simulation series without contrast media. This study did not explore the resulting effects of contrast media on automatic brightness/exposure control.

### 2.6. Procedure scaling

The derived DCs must be multiplied by a clinical dose metric, e.g. reference point air kerma or kerma-area product, in order to estimate the total organ absorbed or detriment-weighted dose for each field and examination as a whole. To obtain this information, a series of measurements were taken on an image-intensifier based Siemens under-table diagnostic fluoroscopy unit. Pulses refer to the system operation in fluoroscopic acquisition mode, while frames refer to the system operation in radiographic spot film acquisition mode. A 19 mm (0.75-inch) aluminum block was placed in the beam under fixed collimation to represent a pediatric patient within the beam (2004), without magnification, and fluoroscopy images (7.5 pulses per second) and radiographic spot film images (single shot) were acquired. Air kerma rate was measured using an external unfors dosimeter^9^9www.raysafe.com/sites/default/files/G21217_Xray_TestEquipment_ProductCatalog-EN-RevG.pdf. equipped with an approximate 2 mm-thick tin backplate, which makes the measured air kerma insensitive to backscattered radiation at angles greater than ±70° (Lordinot *et al* 2025). The dosimeter has an expanded measurement uncertainty of 2.2%, based on recent calibration data at 80 kV. Measurements were taken with the dosimeter placed at the entrance surface of the aluminum phantom and were used to estimate the differences in air kerma for each mode of operation. Based on these measurements, the air kerma delivered from a radiographic spot film is approximately 26 times higher than the air kerma delivered from a single fluoroscopy pulse.

Published values for age-specific kerma-area product quartiles from LaBella *et al* (2024) were used to estimate the dose per radiographic spot film and fluoroscopy pulse. The publication reports a median kerma-area product value for newborn to 1 year-old studies of 0.02 Gy cm^2^. This value was set equal to the total kerma-area product delivered across all radiographic spot films and fluoroscopy time for both the newborn and 1 year old study. The general schema is such that:

\begin{align*}P_{{\mathrm{KA}}}^{{\mathrm{total}}} = P_{{\mathrm{KA}}}^{{\mathrm{fluoro}}} + P_{{\mathrm{KA}}}^{{\mathrm{rad}}}\end{align*}

where $P_{{\mathrm{KA}}}^{{\mathrm{fluoro}}}$ is the total kerma-area product delivered from the fluoroscopy component and $P_{{\mathrm{KA}}}^{{\mathrm{rad}}}$ is the total kerma-area product delivered from the radiographic spot films during an entire UGI series examination. The distribution of kerma-area product between fluoroscopy and radiography components as a function of kerma-area product delivered per delivered x-ray pulse is further shown by

\begin{align*}P_{{\mathrm{KA}}}^{{\mathrm{fluoro}}} = P_{{\mathrm{KA}}}^{{\mathrm{pulse}}} \times t \times R\end{align*}

\begin{align*}P_{{\mathrm{KA}}}^{{\mathrm{rad}}} = P_{{\mathrm{KA}}}^{{\mathrm{pulse}}} \times k \times N\end{align*}

where $P_{{\mathrm{KA}}}^{{\mathrm{pulse}}}$ is the kerma-area product delivered per delivered x-ray pulse, *t* is the total procedure fluoroscopy time in seconds, *R* is the number of pulses per second, *k* is the equivalent delivered pulses per spot film taken, and *N* is the total number of spot films taken during the examination. From this relationship, an estimation of kerma-area product per pulse $\left( {P_{{\mathrm{KA}}}^{{\mathrm{pulse}}}} \right)$ could be made. Normal UGI series procedure outlines for the newborn female require 75 s of fluoroscopy time with 6 spot films; the 1 year-old requires 95 s of fluoroscopy time due to assumed patient motion with 6 spot films. Abnormal UGI series procedure outlines for the newborn female require 80 s of fluoroscopy time with 6 spot films; the 1 year-old requires 95 s of fluoroscopy time due to assumed patient motion with 6 spot films. It was assumed that these studies make use of a 7.5 pulse per second protocol (*R*). By solving for $P_{{\mathrm{KA}}}^{{\mathrm{pulse}}}{ }$ for both the newborn and 1 year-old female phantoms, the resultant dose per pulse was found to be 0.0252 mGy cm^2^, and the dose per frame was 0.655 mGy cm^2^. Tables 7–10 outline the total kerma-area product for the individual components, for fluoroscopy and spot films, for each imaging field across the entirety of the procedure for the newborn and 1 year-old for both normal and abnormal examinations.

**Table 7.** Kerma-area product delivered per imaging field from fluoroscopy and spot film components for a newborn female undergoing a UGI examination resulting in a normal diagnosis.

*P_KA_* (*Gy∙cm^2^*)										
Newborn	Field 1	Field 2	Field 3	Field 4	Field 5	Field 6	Field 7	Field 8	Field 9	Total
Fluoroscopy	9.452 × 10^−04^	9.452 × 10^−04^	1.890 × 10^−03^	1.890 × 10^−03^	1.890 × 10^−03^	1.890 × 10^−03^	1.890 × 10^−03^	1.890 × 10^−03^	9.452 × 10^−04^	1.811 × 10^−02^
Spot film	—	—	—	6.553 × 10^−04^	6.553 × 10^−04^	6.553 × 10^−04^	6.553 × 10^−04^	6.553 × 10^−04^	6.553 × 10^−04^	

**Table 8.** Kerma-area product delivered per imaging field from fluoroscopy and spot film components for a 1 year-old female undergoing a UGI examination resulting in a normal diagnosis.

*P_KA_* (Gy*∙*cm*^2^*)										
1-year-old	Field 1	Field 2	Field 3	Field 4	Field 5	Field 6	Field 7	Field 8	Field 9	Total
Fluoroscopy	1.890 × 10^−03^	9.452 × 10^−04^	1.890 × 10^−03^	1.890 × 10^−03^	2.836 × 10^−03^	2.836 × 10^−03^	1.890 × 10^−03^	2.836 × 10^−03^	9.452 × 10^−04^	2.189 × 10^−02^
Spot film	—	—	—	6.553 × 10^−04^	6.553 × 10^−04^	6.553 × 10^−04^	6.553 × 10^−04^	6.553 × 10^−04^	6.553 × 10^−04^	

**Table 9.** Kerma-area product delivered per imaging field from fluoroscopy and spot film components for a newborn female undergoing a UGI examination resulting in an abnormal diagnosis.

*P*_KA_ (Gy∙cm*^2^*)								
Newborn	Field 1	Field 2	Field 3	Field 4	Field 5	Field 6	Field 7	Total
Fluoroscopy	1.890 × 10^−03^	1.890 × 10^−03^	1.890 × 10^−03^	2.836 × 10^−03^	2.836 × 10^−03^	1.890 × 10^−03^	1.890 × 10^−03^	1.905 × 10^−02^
Spot film	6.553 × 10^−04^	—	6.553 × 10^−04^	6.553 × 10^−04^	6.553 × 10^−04^	6.553 × 10^−04^	6.553 × 10^−04^	

**Table 10.** Kerma-area product delivered per imaging field from fluoroscopy and spot film components for a 1 year-old female undergoing a UGI examination resulting in an abnormal diagnosis.

*P*_KA_ (Gy*∙*cm*^2^*)								
1-year-old	Field 1	Field 2	Field 3	Field 4	Field 5	Field 6	Field 7	Total
Fluoroscopy	1.890 × 10^−03^	1.890 × 10^−03^	1.890 × 10^−03^	2.836 × 10^−03^	3.781 × 10^−03^	2.836 × 10^−03^	2.836 × 10^−03^	2.189 × 10^−02^
Spot film	6.553 × 10^−04^	—	6.553 × 10^−04^	6.553 × 10^−04^	6.553 × 10^−04^	6.553 × 10^−04^	6.553 × 10^−04^	

Absorbed doses for the colon, heart wall, kidneys, lungs, red (active) bone marrow, small intestine wall, spleen, stomach wall, thymus, thyroid, remainder tissues, and detriment-weighted dose were normalized to kerma-area product taken from kerma to an air volume within each simulation multiplied by the area of the field at the air volume location. Organ absorbed and detriment-weighted doses for a complete UGI series examination were calculated using

\begin{align*}{\mathrm{Dos}}{{\mathrm{e}}_i} = \mathop \sum \limits_{f = 1}^n P_{{\mathrm{KA}},f}^{{\mathrm{fluoro}}} \times {\mathrm{DC}}_{i,f}^{{\mathrm{fluoro}}} + P_{{\mathrm{KA}},f}^{{\mathrm{rad}}} \times {\mathrm{DC}}_{i,f}^{{\mathrm{rad}}}\end{align*}

where Dose*_i_* is the total examination dose to organ *i*, $P_{{\mathrm{KA}},f}^{{\mathrm{fluoro}}}$ is the total kerma-area product delivered for field number *f* from the fluoroscopy component, ${\mathrm{DC}}_{i,f}^{{\mathrm{fluoro}}}$ is the DC for organ *i* for field number *f* for x-ray technique factors from the fluoroscopy component, $P_{{\mathrm{KA}},f}^{{\mathrm{rad}}}$ is the total kerma-area product delivered for field number *f* from the radiographic spot film component, and ${\mathrm{DC}}_{i,f}^{{\mathrm{rad}}}$ is the DC for organ *i* for field number *f* for x-ray technique factors from the radiographic spot film component. Organ absorbed and detriment-weighted doses for a single imaging field can be computed by setting *n* equal to *f* in (4).

## 3. Results

Four unique UGI series examinations encompassing two age groups across normal and abnormal disease states complete with fluoroscopic and spot film images were successfully modeled in the Monte Carlo radiation transport simulation software PHITS. From the previously mentioned PHITS simulation parameters, dose tallies for investigated organs that were inside the primary x-ray beam had relative errors of less than 1% while small organs far from the field (e.g. lens of the eye) exceeded 20%. Examination field margins were created from the process outlined in figure 1. Figure 2 provides imaging field data for a newborn female undergoing a UGI series resulting in a normal diagnosis. Figure 3 provides imaging field data for a 1 year-old female undergoing a UGI series resulting in a normal diagnosis. Figure 4 provides imaging field data for a newborn female undergoing a UGI series resulting in an abnormal diagnosis. Figure 5 provides imaging field data for a 1 year-old female undergoing a UGI series resulting in an abnormal diagnosis. Fluoroscopic and spot film image field of view lines are shown as red rectangles while organs that are highlighted in blue represent those that contain contrast media.

LaBella *et al* (2024) reported a median effective dose for patients between the ages of 0 and 1 year-old of 0.031 mSv ± 0.001 mSv while this study found the average detriment-weighted dose for both ages and diagnoses to be 0.030 mSv for simulations containing contrast media and 0.033 mSv for simulations without contrast media. Marshall *et al* (2019) reported organ absorbed DCs for a newborn female phantom for the colon and liver to be 3.46 mGy/Gy cm^2^ and 9.48 mGy/Gy cm^2^, respectively. This study found the organ absorbed DCs for the newborn female ICRP VRCP for a normal UGI series to be 1.83 mGy/Gy cm^2^ and 4.25 mGy/Gy cm^2^ for the colon and liver, respectively. Older studies (Damilakis *et al* 2006, Staton *et al* 2007) have reported effective doses on the order of several mSv, exceeding those reported by LaBella and by this study. Since its initiation in 2007, the Image Gently campaign, in combination with other radiation optimization initiatives, has likely contributed to decreased medical radiation exposure and reduced *P*_KA_ values.

Ratios of organ absorbed and detriment-weighted doses from simulations with contrast media to those doses from simulation without contrast media were computed and are presented in tables 11–14. Relative comparisons are then made to doses without contrast media.

**Table 11.** Ratios of doses to specified organs between simulations with and without contrast media included in the UGI procedure for a newborn female resulting in a normal diagnosis.

Organ	Field 1N^a^	Field 2 N^a^	Field 3 N	Field 4 N	Field 5 N	Field 6 N	Field 7 N	Field 8 N	Field 9 N	Total
Colon	1.00	1.00	0.94	0.87	0.68	0.95	0.93	0.79	0.78	0.88
Ht-wall	1.00	1.00	0.97	1.00	0.91	0.91	0.94	0.83	0.81	0.94
Kidneys	1.00	1.00	0.91	0.86	0.85	0.98	0.97	1.00	1.00	0.98
Lungs	1.00	1.00	0.95	0.97	0.99	0.95	0.95	0.91	0.90	0.98
R-marrow	1.00	1.00	0.97	0.98	0.99	0.97	0.96	0.98	0.98	0.98
SI-wall	1.00	1.00	0.93	0.76	0.66	0.86	0.77	0.88	0.87	0.84
Spleen	1.00	1.00	0.95	1.00	0.98	0.71	0.71	0.99	0.99	0.94
St-wall	1.00	1.00	0.95	0.91	0.71	0.71	0.69	0.74	0.74	0.74
Thymus	1.00	1.00	0.99	1.00	0.87	0.94	0.94	0.87	0.86	0.94
Thyroid	1.00	1.00	0.99	1.00	0.94	0.93	0.93	0.89	0.87	0.98
Remainder	1.00	1.00	0.99	0.98	0.93	0.92	0.90	0.97	0.97	0.96
DW dose	1.00	1.00	0.98	0.98	0.95	0.90	0.88	0.86	0.85	0.92

^a^ **Non contrast containing fields.**

### 3.1. Impact of age

Changes in organ absorbed and detriment-weighted doses per field and organ absorbed and detriment-weighted doses for a total examination between the newborn and 1 year-old female phantoms show similar trends when comparing UGI series simulations with and without contrast media for both normal and abnormal disease states. These findings are demonstrated when referencing tables 11 and 12 which shows that the total procedure absorbed dose to the stomach wall decreased by 26% for both ages and the total procedure detriment-weighted dose decreased by 8% for both ages when barium contrast was incorporated within the Monte Carlo simulations for a normal UGI series examination. Similar findings are shown in tables 13 and 14 where the total procedure absorbed dose to the stomach wall decreased by 13% for the newborn and 16% for the 1 year-old and the total procedure detriment-weighted dose decreased by 6% for the newborn and 7% for the 1 year-old when iodinated contrast media was incorporated in the Monte Carlo simulations for an abnormal UGI series examination. Key differences are noted when comparing the two age groups for field-specific organ absorbed doses for a normal UGI series examination (tables 11 and 12); the spleen dose in Fields 6 N and 7 N decreased by 29% for the newborn female phantom but decreased by 50% for the 1 year-old female phantom, which is likely due to differences in relative organ size and positioning between the two phantoms.

**Table 12.** Ratios of doses to specified organs between simulations with and without contrast media included in the UGI procedure for a 1 year-old female resulting in a normal diagnosis.

Organ	Field 1N^a^	Field 2 N^a^	Field 3 N	Field 4 N	Field 5 N	Field 6 N	Field 7 N	Field 8 N	Field 9 N	Total
Colon	1.00	1.00	0.94	0.77	0.66	0.94	0.92	0.80	0.80	0.88
Ht-wall	1.00	1.00	0.93	0.99	0.90	0.90	0.94	0.77	0.75	0.92
Kidneys	1.00	1.00	0.91	0.84	0.94	0.98	0.97	0.99	0.99	0.98
Lungs	1.00	1.00	0.93	0.97	0.98	0.94	0.94	0.92	0.91	0.97
R-marrow	1.00	1.00	0.97	0.98	0.99	0.97	0.96	0.98	0.97	0.98
SI-wall	1.00	1.00	0.93	0.75	0.77	0.88	0.80	0.86	0.86	0.85
Spleen	1.00	1.00	0.96	0.99	0.96	0.50	0.50	0.96	0.97	0.92
St-wall	1.00	1.00	0.93	0.86	0.71	0.74	0.73	0.71	0.72	0.74
Thymus	1.00	1.00	0.94	0.99	0.84	0.95	0.94	0.86	0.84	0.93
Thyroid	1.00	1.00	0.98	0.97	0.90	0.96	0.96	0.89	0.88	0.96
Remainder	1.00	1.00	0.99	0.97	0.93	0.93	0.91	0.96	0.95	0.95
DW dose	1.00	1.00	0.98	0.96	0.93	0.91	0.90	0.86	0.85	0.92

^a^ **Non contrast containing fields.**

**Table 13.** Ratios of doses to specified organs between simulations with and without contrast media included in the UGI procedure for a newborn female resulting in an abnormal diagnosis.

Organ	Field 1 A^a^	Field 2 A^a^	Field 3 A	Field 4 A	Field 5 A	Field 6 A	Field 7 A	Total
Colon	1.00	1.00	0.96	0.95	0.85	0.84	0.86	0.93
Ht-wall	1.00	1.00	0.94	0.95	0.85	0.83	0.87	0.93
Kidneys	1.00	1.00	0.99	0.98	1.00	1.00	1.00	1.00
Lungs	1.00	1.00	0.94	0.94	0.92	0.91	0.93	0.96
R-marrow	1.00	1.00	0.97	0.96	0.98	0.98	0.99	0.98
SI-wall	1.00	1.00	0.95	0.87	0.97	0.96	0.96	0.95
Spleen	1.00	1.00	0.76	0.77	1.01	1.01	1.00	0.97
St-wall	1.00	1.00	0.82	0.82	0.86	0.86	0.86	0.87
Thymus	1.00	1.00	0.93	0.93	0.88	0.87	0.89	0.93
Thyroid	1.00	1.00	0.90	0.90	0.88	0.87	0.91	0.92
Remainder	1.00	1.00	0.96	0.94	0.99	0.99	0.99	0.98
DW dose	1.00	1.00	0.93	0.92	0.91	0.91	0.92	0.94

^a^ **Non contrast containing fields.**

**Table 14.** Ratios of doses to specified organs between simulations with and without contrast media included in the UGI procedure for a 1 year-old female resulting in an abnormal diagnosis.

Organ	Field 1 A^a^	Field 2 A^a^	Field 3 A	Field 4 A	Field 5 A	Field 6 A	Field 7 A	Total
Colon	1.00	1.00	0.94	0.92	0.86	0.86	0.87	0.91
Ht-wall	1.00	1.00	0.96	0.96	0.81	0.79	0.84	0.92
Kidneys	1.00	1.00	0.98	0.98	1.00	0.99	1.00	0.99
Lungs	1.00	1.00	0.96	0.96	0.94	0.93	0.94	0.96
R-marrow	1.00	1.00	0.98	0.97	0.98	0.98	0.98	0.98
SI-wall	1.00	1.00	0.95	0.88	0.93	0.93	0.94	0.94
Spleen	1.00	1.00	0.54	0.55	0.98	0.99	0.96	0.94
St-wall	1.00	1.00	0.83	0.84	0.80	0.80	0.81	0.84
Thymus	1.00	1.00	0.95	0.95	0.88	0.86	0.90	0.96
Thyroid	1.00	1.00	0.97	0.97	0.87	0.88	0.91	0.95
Remainder	1.00	1.00	0.95	0.94	0.97	0.97	0.97	0.97
DW dose	1.00	1.00	0.94	0.93	0.90	0.89	0.91	0.93

^a^ **Non contrast containing fields**

### 3.2. Organ-specific differences with use of contrast media

Field-specific and total procedure absorbed doses were computed for the colon, heart wall, kidneys, lungs, red (active) bone marrow, small intestine wall, spleen, stomach wall, thymus, thyroid, and remainder tissues as well as a detriment-weighted dose. Tables 11–14 show that across both ages and disease states, the inclusion of contrast media within the contents of an organ generally caused absorbed doses to that organ and those nearby to decrease due to selective absorption of the x-ray beam by the contrast media. This effect was observed for various organs as the contrast media moved through the GI system.

### 3.3. Impact of diagnosis (normal versus abnormal procedure)

Similar trends were observed when comparing consistent imaging fields such as Fields 6 N and 8 N (tables 11 and 12) to Fields 3 A and 5 A (tables 13 and 14), respectively, as they have the same patient geometry and similar contrast media volumes between the two types of examinations (normal versus abnormal). Barium contrast media caused a slightly greater decrease in organ absorbed doses when compared to the equivalent imaging field filled with iodinated contrast media; the organ absorbed dose to the stomach wall decreased by 29% in Field 6 N of the newborn normal UGI series and decreased by 24% in Field 3 A of the newborn abnormal UGI series.

## 4. Discussion

Field-specific organ absorbed doses are shown to decrease by up to 50% when comparing the Monte Carlo simulated UGI series with contrast media to those without. Total procedure organ absorbed doses are shown to decrease by up to 26% for both the newborn and 1 year-old female phantoms. Total procedure detriment weighted doses are shown to decrease by up to 8% for both the newborn and 1-year-old female phantoms.

**Figure 6.**
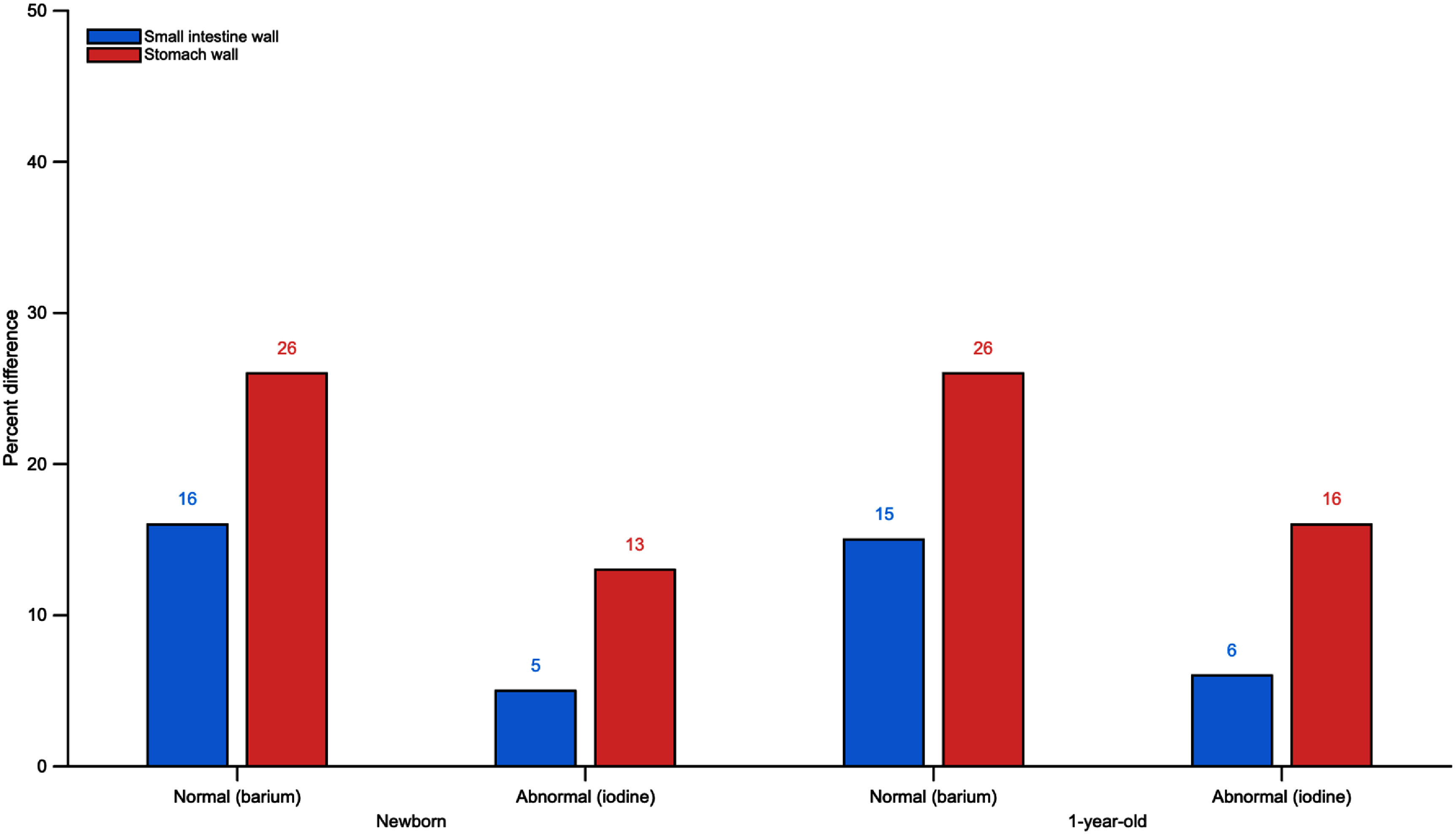
differences in total procedure organ absorbed doses for contrast-containing organs (small intestine wall and stomach wall) in newborn and 1 year-old female phantoms. Percent differences were calculated relative to the non-contrast-containing simulation results.

Similar patterns of change in organ absorbed and detriment-weighted doses were observed between the newborn and 1 year-old female computational phantoms, as well as when comparing normal and abnormal UGI series examinations with matching patient geometry and contrast media volume. Trends for changes in total procedural organ dose in contrast-containing organs, such as the stomach wall and small intestine wall, across both ages and diagnostics conditions are illustrated in figure 6. These differences suggest that similar trends would likely be observed if the same analysis were performed on the male newborn and 1 year-old VRCPs, as these phantoms are essentially identical to the female models except for sex-specific organs, with organ and total body masses being the same at these ages.

Differences in absorbed doses for simulations with and without contrast for the oesophageal wall and oral mucosa were not quantified due to inherent limitations in the voxel phantoms. The entire oesophagus and tongue portion of the oral mucosa had to be replaced or mixed with contrast media as there was no separate oesophageal lumen or oral cavity. Therefore, reported absorbed doses to the oesophageal wall and oral mucosa for Monte Carlo simulations with contrast media were artificially inflated due to the contrast media being modeled in place of these organs. Oesophageal wall and oral mucosa doses were replaced in the contrast-containing simulations with the doses from the simulations without contrast in order to be able to appropriately report detriment-weighted doses. Additionally, contrast media within the stomach and small intestine contents had to be mixed with their original reference contents as the voxel organs are rigid, nondeformable structures. With the advent of mesh-type computational phantoms, organs could be easily deformed to more accurately model the dynamic contrast movement and changing organ volumes that would be expected in a real patient’s motile GI system and potentially provide more accurate dosimetry estimates.

The ICRP effective dose, and the sex-specific detriment-weighted dose, serves as a standardized measure of the approximate dose for patient populations exposed to radiation from medical examinations. The DCs and detriment-weighted doses serve to aid consistent reporting of patient doses associated with these imaging procedures in individual facilities, individual countries or world-wide. Reference organ absorbed and detriment-weighted doses are limited in their applications to patient-specific cumulative doses for UGI series examinations as the ICRP newborn and 1 year-old female reference phantoms and their accompanying reference examination outlines do not account for variations in individual patient anatomy. They also do not account for level of training of clinical staff, operator technique, equipment performance variability, nor cooperation of the infant or child, especially in the case of imaging an abnormal diagnosis.

## 5. Conclusion

This study demonstrates that including barium and iodinated contrast media in Monte Carlo radiation transport simulations is worthwhile as field-specific and total procedure organ absorbed and detriment-weighted doses for newborn and 1 year-old female computational phantoms are shown to decrease for certain organs either containing or in near proximity to contrast media. Barium contrast media in normal UGI series examinations has a similar effect on organ absorbed and detriment-weighted doses as iodinated contrast media in abnormal UGI series examinations. For similar imaging geometries with the inclusion of barium contrast media, field-specific organ absorbed doses decreased up to 50% and field-specific detriment-weighted doses decreased up to 15% with estimates of total procedure doses decreasing up to 26% for the stomach wall. Likewise, the newborn and 1 year-old female computational phantoms exhibited similar trends for matching imaging fields and contrast media volumes. A relative decrease in absorbed dose for organs containing or in near proximity to the contrast media was seen due to the barium and iodine increasing absorption of the primary x-ray beam when compared to reference organ and organ content material compositions. However, one important limitation of the current study is noted. The effects of automatic brightness or exposure control were not modeled or corrected for in this study; consequently, while organ absorbed doses decreased in the simulations when contrast media was present, clinical fluoroscopy systems may compensate for increased attenuation by increasing beam output or technique factors, potentially leading to increased absorbed doses in practice.

## Data Availability

All data that support the findings of this study are included within the article (and any supplementary information files).

## Annex A Elemental compositions for organ regions containing contrast media

**Table A1.** Fractional mass elemental compositions of liquid E-Z-PAQUE® oral suspension contrast mixed with reference mouth (tongue muscle), oesophagus (whole organ), stomach contents, and small intestine contents for the newborn female ICRP VRCP undergoing a UGI series resulting in a normal diagnosis.

Organ	Field	H	C	N	O	Na	P	S	Cl	K	Ca	Fe	Ba	Density (g cm^−3^)
Tongue	1	10.39	10.35	2.43	76.13	0.10	0.10	0.10	0.20	0.20	0.00	0.00	0.00	1.050
	2	10.39	10.35	2.43	76.13	0.10	0.10	0.10	0.20	0.20	0.00	0.00	0.00	1.050
	3	8.97	6.48	1.52	71.46	0.06	0.06	2.17	0.13	0.13	0.00	0.00	9.02	1.174
	4	9.88	8.96	2.10	74.46	0.09	0.09	0.84	0.17	0.17	0.00	0.00	3.23	1.091
	5	10.39	10.35	2.43	76.13	0.10	0.10	0.10	0.20	0.20	0.00	0.00	0.00	1.050
	6	10.39	10.35	2.43	76.13	0.10	0.10	0.10	0.20	0.20	0.00	0.00	0.00	1.050
	7	10.39	10.35	2.43	76.13	0.10	0.10	0.10	0.20	0.20	0.00	0.00	0.00	1.050
	8	10.39	10.35	2.43	76.13	0.10	0.10	0.10	0.20	0.20	0.00	0.00	0.00	1.050
	9	10.39	10.35	2.43	76.13	0.10	0.10	0.10	0.20	0.20	0.00	0.00	0.00	1.050

Oesophagus	1	10.44	15.42	2.55	71.27	0.03	0.03	0.13	0.05	0.05	0.00	0.03	0.00	1.030
	2	10.44	15.42	2.55	71.27	0.03	0.03	0.13	0.05	0.05	0.00	0.03	0.00	1.030
	3	6.60	0.00	0.00	63.65	0.00	0.00	5.63	0.00	0.00	0.00	0.00	24.11	1.464
	4	6.60	0.00	0.00	63.65	0.00	0.00	5.63	0.00	0.00	0.00	0.00	24.11	1.464
	5	6.60	0.00	0.00	63.65	0.00	0.00	5.63	0.00	0.00	0.00	0.00	24.11	1.464
	6	10.44	15.42	2.55	71.27	0.03	0.03	0.13	0.05	0.05	0.00	0.03	0.00	1.030
	7	10.44	15.42	2.55	71.27	0.03	0.03	0.13	0.05	0.05	0.00	0.03	0.00	1.030
	8	10.44	15.42	2.55	71.27	0.03	0.03	0.13	0.05	0.05	0.00	0.03	0.00	1.030
	9	10.44	15.42	2.55	71.27	0.03	0.03	0.13	0.05	0.05	0.00	0.03	0.00	1.030

Stomach contents	1	10.00	22.20	2.20	64.40	0.10	0.20	0.30	0.10	0.40	0.00	0.10	0.00	1.030
	2	10.00	22.20	2.20	64.40	0.10	0.20	0.30	0.10	0.40	0.00	0.10	0.00	1.030
	3	10.00	22.20	2.20	64.40	0.10	0.20	0.30	0.10	0.40	0.00	0.10	0.00	1.030
	4	9.54	19.17	1.90	64.30	0.09	0.17	1.03	0.09	0.35	0.00	0.09	3.29	1.073
	5	7.69	7.09	0.70	63.89	0.03	0.06	3.93	0.03	0.13	0.00	0.03	16.42	1.290
	6	6.60	0.00	0.00	63.65	0.00	0.00	5.63	0.00	0.00	0.00	0.00	24.11	1.464
	7	6.85	1.61	0.16	63.71	0.01	0.01	5.24	0.01	0.03	0.00	0.01	22.37	1.421
	8	6.85	1.61	0.16	63.71	0.01	0.01	5.24	0.01	0.03	0.00	0.01	22.37	1.421
	9	6.85	1.61	0.16	63.71	0.01	0.01	5.24	0.01	0.03	0.00	0.01	22.37	1.421

Small intestine contents	1	10.00	22.20	2.20	64.40	0.10	0.20	0.30	0.10	0.40	0.10	0.00	0.00	1.030
	2	10.00	22.20	2.20	64.40	0.10	0.20	0.30	0.10	0.40	0.10	0.00	0.00	1.030
	3	10.00	22.20	2.20	64.40	0.10	0.20	0.30	0.10	0.40	0.10	0.00	0.00	1.030
	4	10.00	22.20	2.20	64.40	0.10	0.20	0.30	0.10	0.40	0.10	0.00	0.00	1.030
	5	10.00	22.20	2.20	64.40	0.10	0.20	0.30	0.10	0.40	0.10	0.00	0.00	1.030
	6	9.54	19.17	1.90	64.30	0.09	0.17	1.03	0.09	0.35	0.09	0.00	3.29	1.073
	7	9.11	16.38	1.62	64.20	0.07	0.15	1.70	0.07	0.30	0.07	0.00	6.32	1.117
	8	9.11	16.38	1.62	64.20	0.07	0.15	1.70	0.07	0.30	0.07	0.00	6.32	1.117
	9	9.11	16.38	1.62	64.20	0.07	0.15	1.70	0.07	0.30	0.07	0.00	6.32	1.117

**Table A2.** Fractional mass elemental compositions of Liquid E-Z-PAQUE® oral suspension contrast mixed with reference mouth (tongue muscle), oesophagus (whole organ), stomach contents, and small intestine contents for the 1-year-old female ICRP VRCP undergoing a UGI series resulting in a normal diagnosis.

Organ	Field	H	C	N	O	Na	P	S	Cl	K	Ca	Fe	Ba	Density (g cm^−3^)
Tongue	1	10.20	14.26	3.40	71.04	0.10	0.20	0.30	0.10	0.40	0.00	0.00	0.00	1.050
	2	10.20	14.26	3.40	71.04	0.10	0.20	0.30	0.10	0.40	0.00	0.00	0.00	1.050
	3	8.86	8.93	2.13	68.28	0.06	0.13	2.29	0.06	0.25	0.00	0.00	9.02	1.174
	4	9.72	12.35	2.94	70.05	0.09	0.17	1.02	0.09	0.35	0.00	0.00	3.23	1.091
	5	10.20	14.26	3.40	71.04	0.10	0.20	0.30	0.10	0.40	0.00	0.00	0.00	1.050
	6	10.20	14.26	3.40	71.04	0.10	0.20	0.30	0.10	0.40	0.00	0.00	0.00	1.050
	7	10.20	14.26	3.40	71.04	0.10	0.20	0.30	0.10	0.40	0.00	0.00	0.00	1.050
	8	10.20	14.26	3.40	71.04	0.10	0.20	0.30	0.10	0.40	0.00	0.00	0.00	1.050
	9	10.20	14.26	3.40	71.04	0.10	0.20	0.30	0.10	0.40	0.00	0.00	0.00	1.050

Oesophagus	1	10.44	22.74	2.82	63.00	0.10	0.18	0.28	0.22	0.20	0.00	0.02	0.00	1.030
	2	10.44	22.74	2.82	63.00	0.10	0.18	0.28	0.22	0.20	0.00	0.02	0.00	1.030
	3	6.60	0.00	0.00	63.65	0.00	0.00	5.63	0.00	0.00	0.00	0.00	24.11	1.464
	4	6.60	0.00	0.00	63.65	0.00	0.00	5.63	0.00	0.00	0.00	0.00	24.11	1.464
	5	6.60	0.00	0.00	63.65	0.00	0.00	5.63	0.00	0.00	0.00	0.00	24.11	1.464
	6	10.44	22.74	2.82	63.00	0.10	0.18	0.28	0.22	0.20	0.00	0.02	0.00	1.030
	7	10.44	22.74	2.82	63.00	0.10	0.18	0.28	0.22	0.20	0.00	0.02	0.00	1.030
	8	10.44	22.74	2.82	63.00	0.10	0.18	0.28	0.22	0.20	0.00	0.02	0.00	1.030
	9	10.44	22.74	2.82	63.00	0.10	0.18	0.28	0.22	0.20	0.00	0.02	0.00	1.030

Stomach contents	1	10.00	22.20	2.20	64.40	0.10	0.20	0.30	0.10	0.40	0.10	0.00	0.00	1.030
	2	10.00	22.20	2.20	64.40	0.10	0.20	0.30	0.10	0.40	0.10	0.00	0.00	1.030
	3	10.00	22.20	2.20	64.40	0.10	0.20	0.30	0.10	0.40	0.10	0.00	0.00	1.030
	4	9.54	19.17	1.9	64.3	0.09	0.17	1.03	0.09	0.35	0.09	0.00	3.29	1.070
	5	7.69	7.09	0.7	63.89	0.03	0.06	3.93	0.03	0.13	0.03	0.00	16.40	1.290
	6	6.60	0.00	0.00	63.65	0.00	0.00	5.63	0.00	0.00	0.00	0.00	24.11	1.464
	7	6.85	1.61	0.16	63.71	0.01	0.01	5.24	0.01	0.03	0.01	0.00	22.40	1.421
	8	6.85	1.61	0.16	63.71	0.01	0.01	5.24	0.01	0.03	0.01	0.00	22.40	1.421
	9	6.85	1.61	0.16	63.71	0.01	0.01	5.24	0.01	0.03	0.01	0.00	22.40	1.421

Small intestine contents	1	10.00	22.20	2.20	64.40	0.10	0.20	0.30	0.10	0.40	0.1	0.00	0.00	1.030
	2	10.00	22.20	2.20	64.40	0.10	0.20	0.30	0.10	0.40	0.1	0.00	0.00	1.030
	3	10.00	22.20	2.20	64.40	0.10	0.20	0.30	0.10	0.40	0.1	0.00	0.00	1.030
	4	10.00	22.20	2.20	64.40	0.10	0.20	0.30	0.10	0.40	0.1	0.00	0.00	1.030
	5	10.00	22.20	2.20	64.40	0.10	0.20	0.30	0.10	0.40	0.1	0.00	0.00	1.030
	6	9.54	19.17	1.9	64.3	0.09	0.17	1.03	0.09	0.35	0.09	0.00	3.29	1.073
	7	9.11	16.38	1.62	64.2	0.07	0.15	1.7	0.07	0.3	0.07	0.00	6.32	1.117
	8	9.11	16.38	1.62	64.2	0.07	0.15	1.7	0.07	0.3	0.07	0.00	6.32	1.117
	9	9.11	16.38	1.62	64.2	0.07	0.15	1.7	0.07	0.3	0.07	0.00	6.32	1.117

**Table A3.** Fractional mass elemental compositions of OMNIPAQUE™ Injection contrast mixed with reference mouth (tongue muscle), oesophagus (whole organ), stomach contents, and small intestine contents for the newborn female ICRP VRCP undergoing a UGI procedure resulting in an abnormal diagnosis.

Organ	Field	H	C	N	O	Na	P	S	Cl	K	Ca	I	Density (g cm^−3^)
Stomach contents	1	10.00	22.20	2.20	64.40	0.10	0.20	0.30	0.10	0.40	0.10	0.00	1.030
	2	10.00	22.20	2.20	64.40	0.10	0.20	0.30	0.10	0.40	0.10	0.00	1.030
	3	9.77	4.78	0.93	76.11	0.00	0.00	0.00	0.00	0.00	0.00	8.41	1.009
	4	9.82	8.32	1.19	73.73	0.02	0.04	0.06	0.02	0.08	0.02	6.70	1.013
	5	9.82	8.32	1.19	73.73	0.02	0.04	0.06	0.02	0.08	0.02	6.70	1.013
	6	9.82	8.32	1.19	73.73	0.02	0.04	0.06	0.02	0.08	0.02	6.70	1.013
	7	9.82	8.32	1.19	73.73	0.02	0.04	0.06	0.02	0.08	0.02	6.70	1.013

Small bowel contents	1	10.00	22.20	2.20	64.40	0.10	0.20	0.30	0.10	0.40	0.10	0.00	1.030
	2	10.00	22.20	2.20	64.40	0.10	0.20	0.30	0.10	0.40	0.10	0.00	1.030
	3	9.98	20.49	2.08	65.55	0.09	0.18	0.27	0.09	0.36	0.09	0.83	1.028
	4	9.90	17.00	1.80	67.80	0.00	0.10	0.20	0.00	0.20	0.00	2.40	1.024
	5	9.90	17.00	1.80	67.80	0.00	0.10	0.20	0.00	0.20	0.00	2.40	1.024
	6	9.90	17.00	1.80	67.80	0.00	0.10	0.20	0.00	0.20	0.00	2.40	1.024
	7	9.90	17.00	1.80	67.80	0.00	0.10	0.20	0.00	0.20	0.00	2.40	1.024

**Table A4.** Fractional mass elemental compositions of OMNIPAQUE™ Injection contrast mixed with reference mouth (tongue muscle), oesophagus (whole organ), stomach contents, and small intestine contents for the 1-year-old female ICRP VRCP undergoing a UGI procedure resulting in an abnormal diagnosis.

Organ	Field	H	C	N	O	Na	P	S	Cl	K	Ca	I	Density (g cm^−3^)
Stomach contents	1	10.00	22.20	2.20	64.40	0.10	0.20	0.30	0.10	0.40	0.10	0.00	1.030
	2	10.00	22.20	2.20	64.40	0.10	0.20	0.30	0.10	0.40	0.10	0.00	1.030
	3	9.77	4.78	0.93	76.11	0.00	0.00	0.00	0.00	0.00	0.00	8.41	1.009
	4	9.82	8.32	1.19	73.73	0.02	0.04	0.06	0.02	0.08	0.02	6.70	1.013
	5	9.82	8.32	1.19	73.73	0.02	0.04	0.06	0.02	0.08	0.02	6.70	1.013
	6	9.82	8.32	1.19	73.73	0.02	0.04	0.06	0.02	0.08	0.02	6.70	1.013
	7	9.82	8.32	1.19	73.73	0.02	0.04	0.06	0.02	0.08	0.02	6.70	1.013

Small bowel contents	1	10.00	22.20	2.20	64.40	0.10	0.20	0.30	0.10	0.40	0.10	0.00	1.030
	2	10.00	22.20	2.20	64.40	0.10	0.20	0.30	0.10	0.40	0.10	0.00	1.030
	3	9.98	20.49	2.08	65.55	0.09	0.18	0.27	0.09	0.36	0.09	0.83	1.028
	4	9.93	17.05	1.82	67.86	0.07	0.14	0.21	0.07	0.28	0.07	2.49	1.024
	5	9.93	17.05	1.82	67.86	0.07	0.14	0.21	0.07	0.28	0.07	2.49	1.024
	6	9.93	17.05	1.82	67.86	0.07	0.14	0.21	0.07	0.28	0.07	2.49	1.024
	7	9.93	17.05	1.82	67.86	0.07	0.14	0.21	0.07	0.28	0.07	2.49	1.024
